# Impact of surgical approach on progress of disease by type of histology in stage IA endometrial cancer: a matched-pair analysis

**DOI:** 10.1186/s12893-023-02299-7

**Published:** 2024-01-03

**Authors:** Huixian Miao, Lin Zhang, Yi Jiang, Yicong Wan, Lin Yuan, Wenjun Cheng

**Affiliations:** Department of Gynecology, Jiangsu Province Hospital, the First Affiliated Hospital With Nanjing Medical University, No. 300 Guangzhou Road, Nanjing, 210029 Jiangsu China

**Keywords:** Endometrial carcinoma, Histologic type, Minimal invasive surgery, Open surgery, Prognosis

## Abstract

**Background:**

To compare the impact of surgical approach on progression free survival (PFS) stratified by histologic type in women diagnosed with stage IA endometrial cancer.

**Methods:**

Myometrial invasion is classified into no myometrial invasion, <50% and ≥50%, with only no myometrial invasion and <50% are included in stage IA patients. A retrospective study is designed by collecting data from women diagnosed as stage IA endometrial cancer from January 2010 to December 2019 in a tertiary hospital. A propensity score is conducted for 1:1 matching in the low-risk histologic patients. Progression free survival and disease-specific survival data are evaluated by the Kaplan–Meier method and compared by the log-rank test in both the whole population and the matched-pair groups. A sub-group analysis is performed to figure out risk factors associated with the effect of surgical approach on PFS and disease-specific survival (DSS).

**Results:**

534 (84.49%) low-risk histologic endometrial cancer women, with 389 (72.85%) operated by minimally invasive surgery and 145 (27.15%) by open approach, and 98 (15.51%) high-risk histology, with 71 (72.45%) by laparoscopy and 27 (27.55%) by open surgery, are included. Compared to open surgery, laparoscopy results in lower progression free survival in low-risk patients before and after matching (*p* = 0.039 and *p* = 0.033, respectively), but shows no difference in high-risk patients (*p* = 0.519). Myometrial invasion is associated with lower progression free survival in laparoscopy in low-risk histology (*p* = 0.027).

**Conclusion:**

Surgical approaches influence progression free survival in stage IA low-risk histologic diseases, especially in those with myometrial invasion, but not in high-risk histologic endometrial cancer.

**Supplementary Information:**

The online version contains supplementary material available at 10.1186/s12893-023-02299-7.

## Background

Endometrial cancer (EC) is one of the most prevalent gynecological malignancies and the second leading cause of gynecological cancer death in the developed countries and in China [1]. Despite early diagnosis in nearly two-thirds of EC patients, recurrence remains a common occurrence, with 21% of cases involving regional diseases and 8% involving distant diseases [2]. As reported, an estimate number of more than 17,100 women die of this malignancy per year in our country, whose incidence and mortality has been rising in the past years [3].

EC can be classified into two types based on pathogenic characteristics [4]. Type 1, which is related to relative excess of exposure to estrogen, includes grade 1–2 endometrioid histology, characterized by favorable histopathological features and therefore presents an optimum outcome. On the contrary, type 2, which has little correlation with prior relative excess estrogen exposure, includes grade 3 endometrioid adenocarcinoma (G3E), papillary serous carcinoma (PS), clear cell carcinoma (CC) and carcinosarcoma (CS), often invade outside the uterine, and has a worse outcome, and therefore type 2 is also known as high-risk histologic EC [5, 6].

Many studies have demonstrated the oncologic outcomes including 5-year survival rate and progression free survival (PFS) for EC are comparable between minimally invasive surgery (MIS) and open surgery approach (OP), and MIS is associated with a shorter hospital stay, less perioperative complications and better quality of life [7, 8], which allows MIS to be a preferrable route to accomplish comprehensive surgery in EC, especially in stage I patients [9, 10]. However, a recent randomized trial has reported in 2018 that in early-stage cervical carcinoma, women underwent MIS suffer an increased risk of relapse and death compared to OP [11, 12]. These finding have prompted questions about whether MIS should be adopted as the gold standard for treating gynecologic cancers, including endometrial cancer.

Few studies have enrolled only a small sample of type 2 EC or even have not included type 2 EC [10]. This study, however, enrolled endometrial cancer with all types of histology in Asian patients, is aimed to evaluate the impact of surgical route on progression free survival stratified by histologic type in women diagnosed with stage IA EC, and to determine the risk factors related to its oncologic outcomes.

## Materials and methods

### Ethic statement

This retrospective study was approved by the Ethics Committee of the First Affiliated Hospital of Nanjing Medical University (2020-MD-371) and was conducted in accordance with the Helsinki Declaration. Informed consent was waived by the Ethics Committee of the First Affiliated Hospital of Nanjing Medical University due to the retrospective nature of our project.

### Study design and population

This retrospective study was conducted in a cohort of women pathologically confirmed with stage IA endometrial cancer between January 2010 and December 2019 at a tertiary hospital in China. Patients who underwent hysterectomy and was known of surgical route (minimally invasive or open) were elected for further analysis. Data including age, parity, medical complications (hypertension, diabetes), menopause, body mass index (BMI), clinical symptoms, histologic type, surgical staging according to The International Federation of Gynecology and Obstetrics (FIGO) EC classification, adjuvant treatment was extracted from the electronic medical records.

In our clinic, every EC patient would be evaluated through CT, MRI or ultrasound before staging surgery. For apparent early-stage patients, we would prefer MIS since the MIS was recommended in these patients. However, the final surgical approach was determined by various factors, such as patients’ age, weight, estimated surgical time and most importantly, their incomes and medical insurance.

Adjuvant therapies were recommended for patients with high grade tumors or with myometrial invasion, according to the guidelines and expert consensus in our country. Moreover, other risk factors might also influence the decision of adjuvant therapy, including LVSI, patients’ age, tumor volume, depth of invasion, and last but not least, patients’ wish.

### Outcomes

Primary outcome is progression free survival (PFS), defined as the time between treatment aimed at shrinking or controlling cancer, and signs that it has started to grow again. Secondary outcomes were disease specific survival (DSS), ascites cytology and recurrence site. DSS is defined as the period between surgical staging and death resulted from the cancer,

### Matched-pair model

A statistical model using matched pairs (1:1) was conducted in our study between the groups, which might be the best prediction of a clinical trial in the retrospective studies and could avoid the possible bias in patient selection. When electing the models, we selected variables that could have an impact on patient survival in order to homogenize both study groups according to the NCCN guidelines and the univariate analysis in our cohort (Supplementary Table S1), including patients’ age, body weight, complicated with hypertension, diabetes, lymphadenectomy, adjuvant therapy, tumor grade and LVSI.

### Statistical analysis

Patient characteristics were summarized by percentage and frequency for categorical variables, and by mean and standard error/median and range for continuous variables. The distribution of categorical variables was compared with chi-square test or Fisher’s exact test and continuous variables with the t test or Mann Whitney U test. The PFS and DSS were studied using the Kaplan–Meier method. The equality of survival curves was tested using the log rank test. Cox regression analysis was used to compare the cohorts, and to assess the factors related to DFS and PFS, by calculating the hazard ratio (HR) with 95% confidence interval (CI). The statistical analyses were two-sided and *p*-values < 0.05 were considered statistically significant. The statistical calculations were carried out by SPSS version 26.0 (SPSS, IBM Corp, Armonk, NY).

## Results

### Whole sample: MIS was associated with reduced PFS in stage IA low-risk histologic EC

Out of 665 women pathologically confirmed with primary stage IA EC and underwent hysterectomy in our hospital, 33 women lost follow-ups and a total of 632 patients were enrolled in the final analysis, of whom, 534 (84.49%) were diagnosed as low-risk histologic type EC and 98 (15.51%) as high-risk histology **(**Table 1). The MIS procedure in our study were all performed through the conventional laparoscopy. A total of 18 (2.85%) patients relapsed in our study, and 16 (3.48%) in MIS and 2 (1.16%) in OP, respectively (*p* = 0.197).

**Table 1:** Demographics and pathology results in women with stage IA endometrial cancer

Variable	Low risk (*n* = 534)			High risk (*n* = 98)		
	**MIS (*n* = 389)**	**OP(*n* = 145)**	***P* value**	**MIS (*n* = 71)**	**OP(*n* = 27)**	***P* value**
Age(years)	51.89 ± 8.99	56.37 ± 9.19	<0.001	56.07 ± 9.27	60.19 ± 9.71	0.056
BMI>24(kg/m^2^)	117(30.08%)	37(25.52%)	0.301	27(38.03%)	7(25.93%)	0.280
Arterial hypertension	127(32.65%)	46(31.72%)	0.839	26(36.62%)	9(33.33%)	0.762
Diabetes mellitus	50(12.85%)	22(15.17%)	0.485	11(15.49%)	3(11.11%)	0.571
Menopause	190(48.84%)	93(64.14%)	0.002	50(70.42%)	22(81.48%)	0.268
Nulliparity	26(6.68%)	7(4.83%)	0.428	3(4.22%)	1(3.70%)	0.906
Lymphadenectomy			0.001			0.239
Sentinel pelvic	43(15.99%)	0(0.00%)		2(3.23%)	0(0.00%)	
Systemic pelvic	58(21.57%)	39(47.56%)		8(12.90%)	13(15.66%)	
Systemic pelvic and para-aortic	168(62.45%)	43(52.44%)		52(83.87%)	70(84.34%)	
Radiotherapy	22(5.66%)	5(3.45%)	0.300	11(15.49%)	7(25.93%)	0.246
Chemotherapy	100(25.71%)	17(11.72%)	0.001	51(71.83%)	15(55.56%)	0.125
MI			0.071			0.645
None	151(38.82%)	44(30.34%)		19(27.76%)	6(22.22%)	
<1/2	238(61.18%)	101(69.66%)		52(73.24%)	21(77.78%)	
Grade			0.433			0.400
G1	173(44.47%)	59(40.69%)		–	–	
G2	216(55.53%)	86(59.31%)		–	–	
G3 Endometrioid	–	–		46(64.79%)	15(55.56%)	
Non-endometrioid	–	–		25(35.21%)	12(44.44%)	
Positive LVSI	17(4.37%)	5(3.45%)	0.634	12(16.90%)	4(14.81%)	0.801
Positive peritoneal cytology	55(14.14%)	8(5.52%)	0.006	12(17.65%)	2(7.41%)	0.343
Recurrence	7(1.80%)	0(0.00%)	0.198	9(12.68%)	2(7.41%)	0.443
Death	2(0.51%)	0(0.00%)	>0.99	2(2.82%)	0(0.00%)	>0.99
Length of follow-up (months)	52.88 ± 24.75	83.96 ± 33.95	<0.001	47.96 ± 21.66	65.63 ± 40.28	0.006

**Fig. 1:**
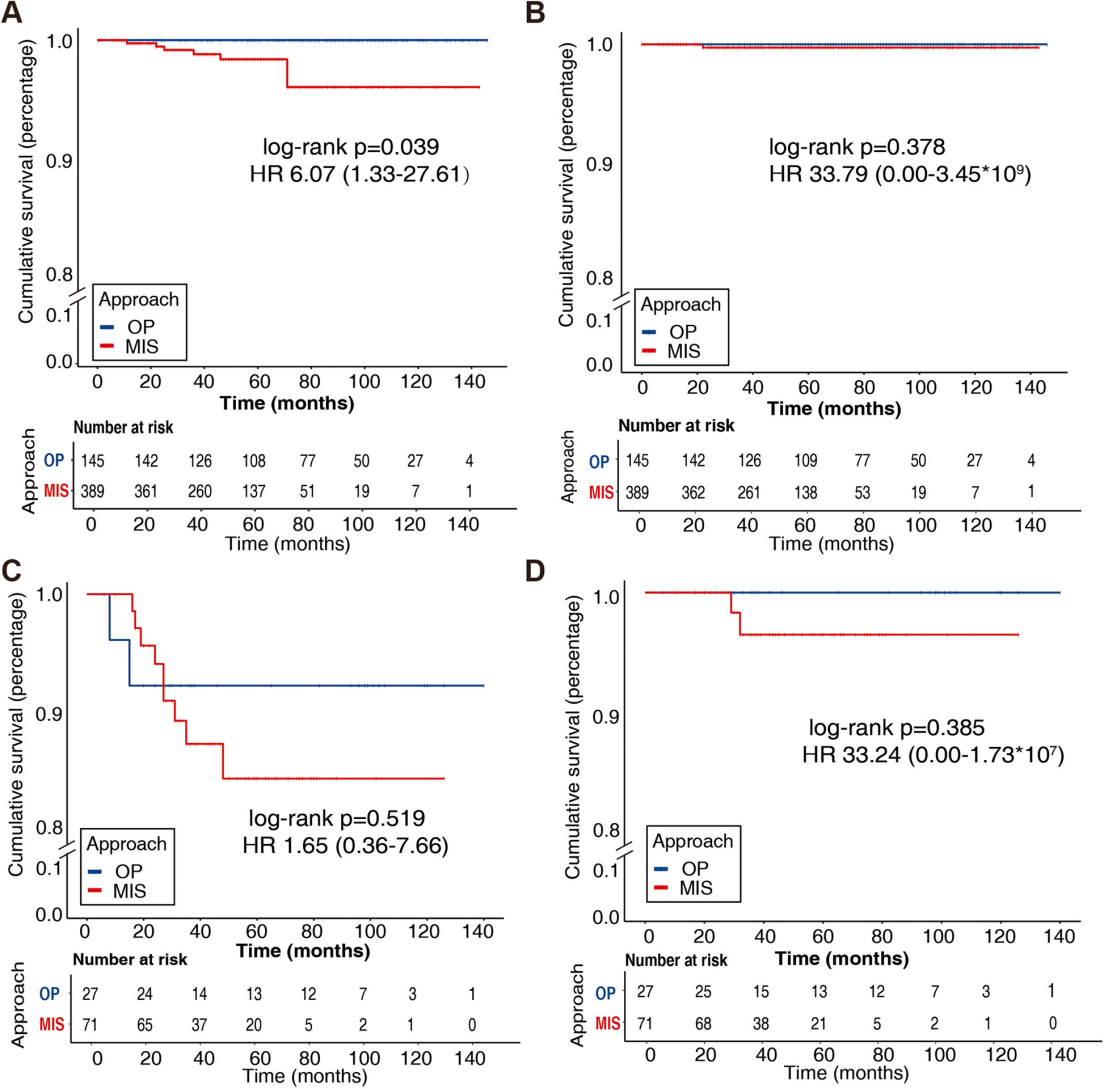
Kaplan–Meier curves for the whole population. (**A**) Progression free survival and (**B**) disease-specific survival in low-risk histologic EC. (**C**) Progression free survival and (**D**) disease-specific survival in high-risk histologic EC

In low-risk histologic EC, open surgical approach (OP) was performed in 145 (27.15%) cases and 389 (72.85%) cases were operated through minimally invasive route (MIS). The general characteristics were shown in Table 1. Compared to OP, women underwent laparoscopy were younger (51.89 ± 8.99 vs 56.39 ± 9.19, *p* < 0.001), had a lower ratio of myometrial invasion (60.41% vs 69.66%, *p* = 0.049), but suffered a higher proportion of positive peritoneal cytology (14.14% vs 5.52%) and chemotherapy (25.71% vs 11.72%, *p* = 0.001). 7 (1.8%) patients relapses and 2 (0.51%) died due to EC in MIS while no recurrence and death occurred in OP after a mean ± SD follow-up of 61.32 ± 30.80 months. Kaplan–Meier curves (Fig. 1A, B) showed a longer progression free survival (PFS) in the open surgery group [log-rank *p* = 0.039, HR 6.07(1.33–27.61)], while disease-specific survival (DSS) was similar in the two groups [log-rank *p* = 0.378, HR 33.79(0.00–3.45*10^9^)].

In 98 women diagnosed as high-risk histologic EC, 71 (72.45%) of them underwent laparoscopy and 27 (27.55%) received open surgery, whose baseline characteristics were of no statistically significance (Table 1). After a mean ± SD follow-up of 52.83 ± 28.92 months, 9 (12.68%) patients suffered recurrence and 2 (2.82%) died due to EC in MIS, along with 2(7.41%) women recurred and none of them died in OP, in which there was no statistically difference. On the other hand, Kaplan–Meier curves also indicated a comparable PFS [log-rank *p* = 0.519, HR 1.65(0.36–7.66)] and DSS [log-rank *p* = 0.385, HR 33.24(0.00–1.73*10^7^)] in the two groups (Fig. 1C, D). Above all, the route of surgery was believed to have no influence on survival in high-risk histologic EC women.

### Sub-analysis: laparoscopy decreased PFS in stage IA low-risk histologic EC with MI

Myometrial invasion (MI), age older than 60 years old and lymph-vascular space invasion (LVSI) were considered as prognostic factors for stage IA EC patients, and hence we analyzed the impact of surgical approach on clinical outcomes stratified by these factors in low-risk histologic EC.

**Fig. 2:**
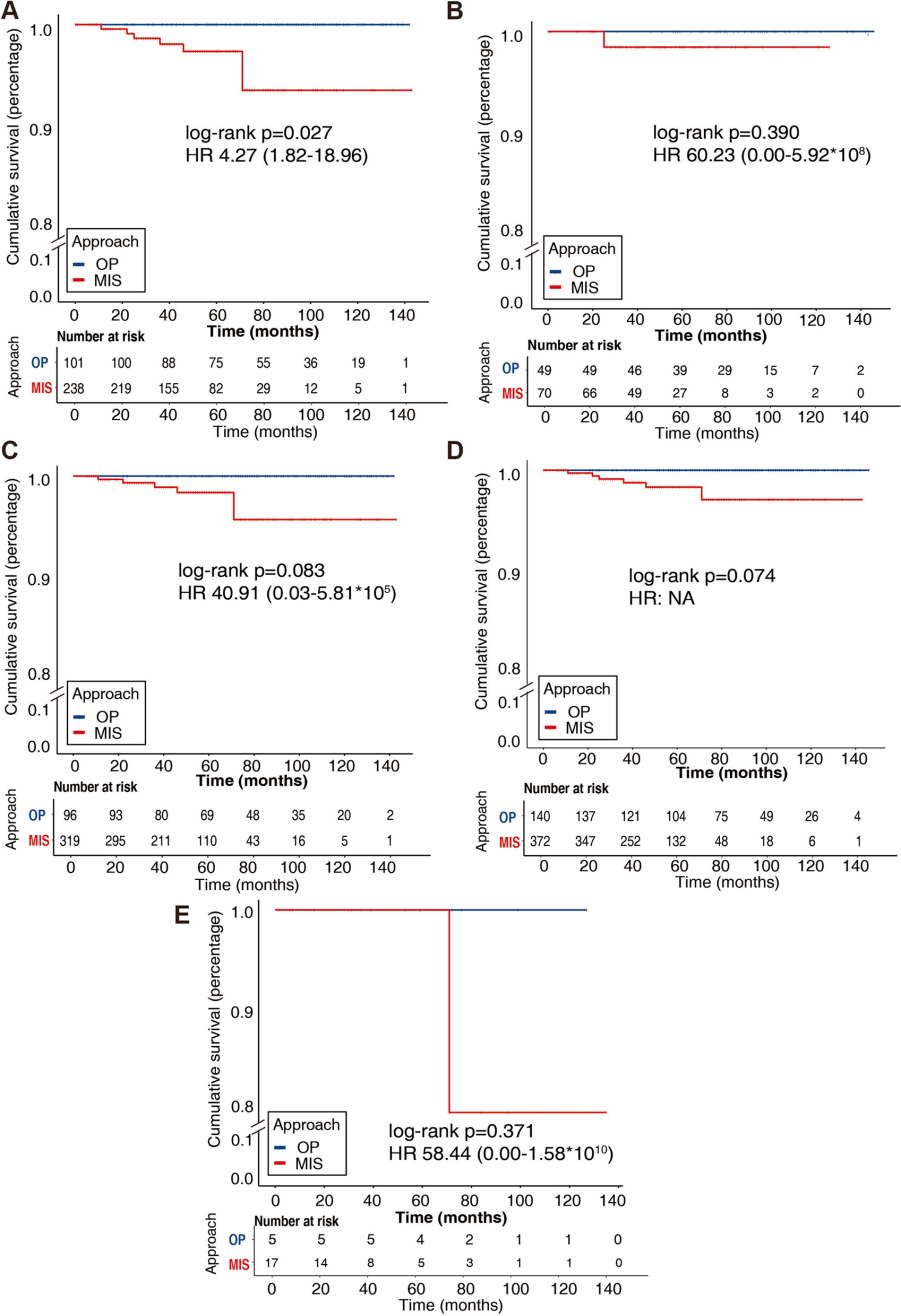
Kaplan–Meier curves of progression free survival in subgroup of low-risk histologic EC: (**A**)in EC with myometrial invasion, (**B**) in women younger than 60 years old, (**C**) in women older than 60 years old, (**D**) in EC without LVSI, (**E**) in EC with LVSI

As shown in Table 1, in 534 low-risk histologic stage IA EC women, 195 were histologically diagnosed with no myometrial invasion and 339 cases were with invasion of the myometrium. Not surprisingly, there was no recurrence (0/195) and deaths (0/195) in patients without MI whatever surgical type. In EC with MI, 238 cases underwent MIS and 101 received OP. The baselines were available in Supplementary Table S2. Compared to OP, women in MIS group were younger (53.40 ± 8.87 versus 57.04 ± 9.40 years old, *p* = 0.001), had a higher rate of positive peritoneal wash (15.97% versus 4.95%, *p* = 0.005) and in turn a higher proportion of chemotherapy (36.55% versus 12.87%, *p* < 0.001). After a mean ± SD follow-up of 62.12 ± 31.35 months, 7 (2.94%) recurrence and 1 (0.42%) death eventually occurred in the MIS group, and no relapse (0.00%) and deaths (0.00%) hit on the OP group, although it was of no statistically (*p* = 0.108 and *p* > 0.99, respectively). However, Kaplan–Meier curves in Fig. 2(A) exhibited that the women benefit from a longer PFS in the OP than those in the MIS group [log-rank *p* = 0.027, HR 4.27(1.82–18.96)].

When it comes to age at diagnose, as shown in Table 1, in low-risk histologic EC, 119 women were older than 60 years old and 415 were younger than 60 years old, whose characteristics were summarized in Supplementary Table S3**.** In women older than 60 years old, only 1 (1.43%) patient suffered relapse in MIS along with none occurred in OP (*p* > 0.99), which was consistent with PFS [log-rank *p* = 0.390, HR 60.23(0.00–5.92*10^8^)] shown in the Kaplan–Meier curves (Fig. 2B). In women younger than 60 years old, 6 (1.88%) recurrence occurred in MIS and none (0.00%) in OP, although there was of no statistical difference (*p* = 0.344), which was the same as PFS shown in Fig. 2C [log-rank *p* = 0.083, HR 40.91(0.03–5.81*10^5^)].

As for the status of LVSI, most of our low-risk histologic EC patients (95.88%) were histologically confirmed without LVSI, whose characteristics were available in Supplementary Table S4. As Kaplan–Meier curves indicated in (Fig. 2D, E), significant survival difference was failed to be observed between the MIS and OP, no matter if the tumor cells invaded into lymph-vascular space. Overall, MI turned out a possible risk factor of relapse associated with surgical approach in low-risk histologic EC women.

### A paired analysis: surgical approach was a prognostic factor in stage IA low-risk histologic EC, especially in EC with MI

**Fig. 3:**
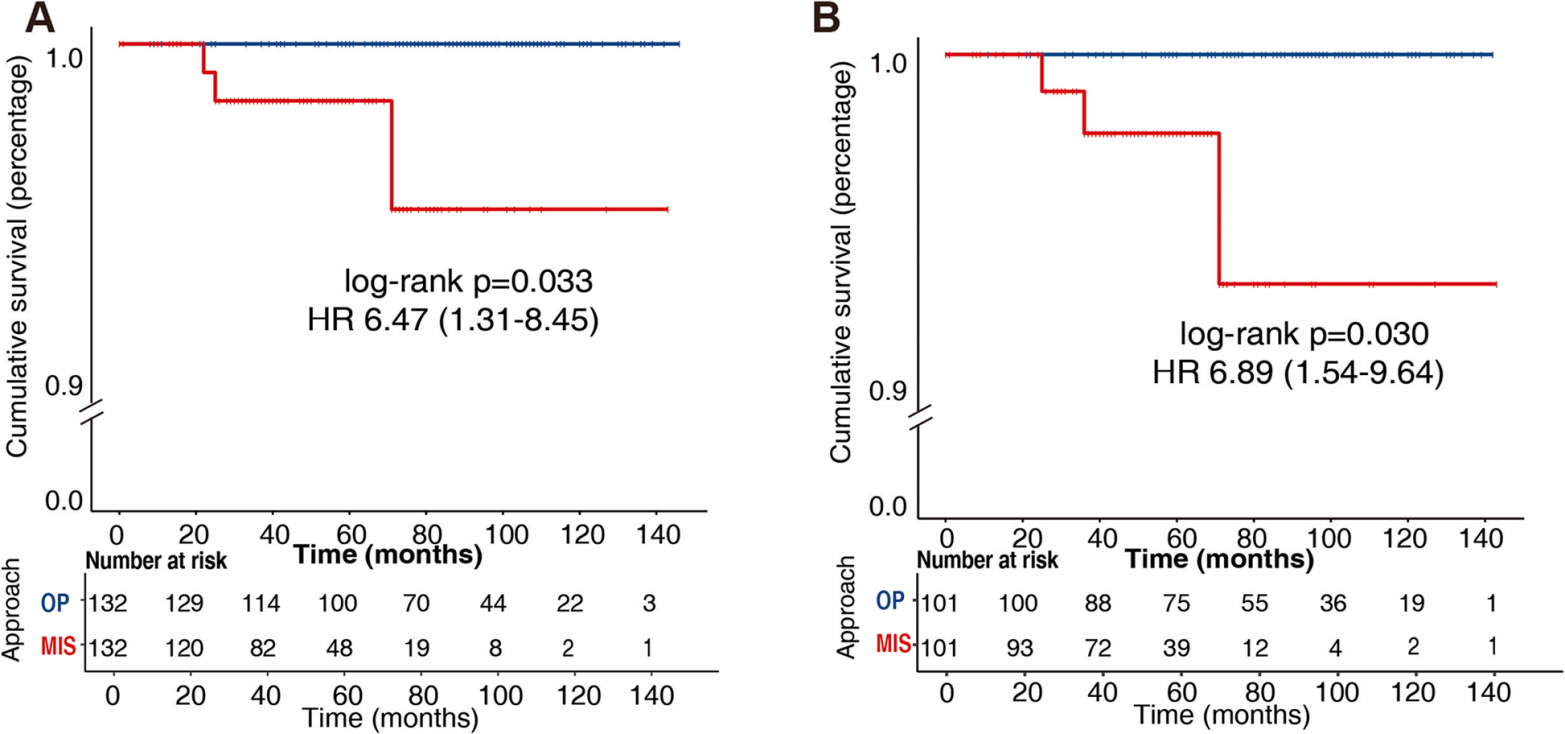
Kaplan–Meier curves of progression free survival in low-risk histologic after matching: (**A**) in all low-risk histologic EC; (**B**) in low-risk histologic EC with MI

Since the baselines were not well-matched between the MIS and OP group in stage IA low-risk histologic EC (Table 1), we conducted a matched-pair (1:1) statistic model to eliminate the deviation of characteristics between the MIS and OP group in stage IA low-risk EC women, and 264 women were enrolled in the final analysis (132 in each group). The matched baselines were available in Table 2, which was well-balanced between the two groups. Eventually, 3 (2.27%) recurrence occurred in the MIS group with none happened (0.00%) in the OP group. Kaplan–Meier curves in Fig. 3A also showed that women underwent open surgery acquired a longer PFS [log-rank *p* = 0.033, HR 6.47(1.31–8.45)], which in turn proved that MIS was a prognostic factor in stage IA low-risk EC women.

**Table 2:** Demographics and pathology results in low-risk histologic stage IA EC after matching

Variable	Low-risk histology			Low-risk histology with MI		
	**MIS (*n* = 132)**	**OP(*n* = 132)**	***P* value**	**MIS (*n* = 101)**	**OP(*n* = 101)**	***P* value**
Age ≥ 60yrs	38(28.79%)	38(28.79%)	>0.99	38(37.62%)	38(37.62%)	>0.99
BMI>24(kg/m^2^)	20(15.15%)	20(15.15%)	>0.99	21(20.79%)	21(20.79%)	>0.99
Arterial hypertension	45(34.09%)	39(29.55%)	0.428	32(31.68%)	32(31.68%)	>0.99
Diabetes mellitus	13(9.85%)	19(14.39%)	0.258	15(14.85%)	15(14.85%)	>0.99
Menopause	66(50.00%)	80(60.61%)	0.083	69(68.32%)	66(65.35%)	0.654
Nulliparity	14(10.61%)	6(4.55%)	0.063	4(3.96%)	8(7.92%)	0.234
Lymphadenectomy	75(56.82%)	76(57.58%)	0.901	63(62.38%)	55(54.46%)	0.253
Radiotherapy	3(2.27%)	3(2.27%)	>0.99	4(3.96%)	4(3.96%)	>0.99
Chemotherapy	15(11.36%)	15(11.36%)	>0.99	13(12.87%)	13(12.87%)	>0.99
MI	89(67.42%)	89(67.42%)	>0.99	–	–	–
Grade			>0.99			>0.99
G1	60(45.45%)	56(42.42%)		27(26.73%)	25(24.75%)	
G2	72(54.55%)	76(57.58%)		74(73.27%)	76(75.25%)	
Positive LVSI	3(2.27%)	3(2.27%)	>0.99	7(6.93%)	4(3.96%)	0.352
Positive peritoneal cytology	15(11.36%)	9(6.82%)	0.199	6(5.94%)	13(12.87%)	0.147
Recurrence	3(2.27%)	0(0.00%)	0.246	0(0.00%)	3(2.97%)	0.246
Death	2(1.52%)	0(0.00%)	0.498	0(0.00%)	0(0.00%)	–
Length of follow-up (months)	52.08 ± 26.25	82.91 ± 33.58	<0.001	84.68 ± 33.17	53.94 ± 25.06	<0.001

Next, a total of 101 matched pairs (202 women) were included to verify the impact of surgical approach on survival in stage IA low-risk EC with MI, both of whom were similar in all variables (Table 2**)**, except for the length of follow-ups. Compared to the OP group, though the length of follow-up was shorter in the MIS group (53.94 ± 25.06 vs 84.68 ± 33.17 months, *p* < 0.001), more recurrence occurred (2.97% vs 0.00%, *p* = 0.247) and the PFS was shorter [log-rank *p* = 0.030, HR 6.89(1.54–9.64)] in the MIS group (Fig. 3B). Besides, we performed a multivariate COX regression analysis in the low-risk EC to correct the potential bias (Supplementary Table S5), in which we selected the significant factors in the univariate COX regression model (Supplementary Table S1). As shown in Table S2, in the multivariate analysis, the surgical approach was also indicated as an independent prognostic factor in the low-risk EC [*p* value = 0.040, HR 5.58(1.23–25.25)]. Hence, laparoscopy was a risk factor of relapse for stage IA low-risk histologic EC, especially for those with MI.

## Discussion

The influence of surgical approach on oncological outcomes in patients with endometrial cancer is investigated in this study. Our study finds that, compared to open surgery approach (OP), the minimally invasive surgery (MIS) would deteriorate the progression free survival (PFS) in patients with early-stage low-risk histologic endometrial cancer (EC) but not in high-risk histologic EC, and myometrial invasion (MI) is identified as a risk factor for these patients. Specially, although no difference is detected in the disease-specific survival (DSS), a significant difference is also observed in PFS in favor of OP after a matched-pair analysis.

Comprehensive surgery is now the primary treatment for apparent early-stage EC. Two randomized trials, which enrolled both type 1 and type 2 histology, have supported that MIS is as oncological safe as OP, meanwhile has faster recovery and fewer perioperative complications than OP in stage I endometrial cancer treatment [7, 10]. However, the safety of MIS in gynecologic cancers has been again called into question since the publication of LACC trial, a landmark phase III study, in the New England Journal of Medicine [11], which highlighted that a significantly higher relapse and death as well as a lower 3-year survival rate associated with MIS compared to OP in women with cervical cancer from 2 to 4 cm. This has led a reconsideration of whether MIS is superior to OP in the EC management, and which patients are suitable for MIS, especially in early-stage EC. Several retrospective studies have also been conducted to explore the noninferiority of MIS compared to OP in terms of clinical outcomes in early-stage EC [13–17]. The US Gynecologic Oncology Group’s (GOG) LAP2 trial and the Laparoscopic Approach to Cancer of the Endometrium (LACE) trial were the two most important randomized trials to evaluate the outcomes between OP and MIS in EC. LAP2, enrolled 2616 clinical stage I-IIA (FIGO 1988 standards) EC patients with all types of cancer histology, reported that laparoscopic surgical staging for EC was feasible due to its short-term safety and length-of-stay in 2009 [7]. However, their follow-up results in 2012 demonstrated that MIS had an estimated 3-year recurrence rate of 11.4% compared with 10.2% for OP, suggested that MIS was not as good as OP in terms of recurrent disease [18], which was similar to our findings. The LACE trial, enrolled 760 stage I (FIGO 1988) EC patients, reported that MIS was equivalent to OP in disease-free survival and overall survival at 4.5 years [10], supporting the use of MIS in stage I patients. Different from our study, their cohort baseline characteristics were quite different, for example, up to 60 percent of their patients were with obesity, nearly half of their patients were elderly (over 65 years old), quite a few (~ 20%) individuals in LACE trial were finally diagnosed with advanced stage diseases, and notably, they did not provide the subgroup survival analysis in their patients. As for our study, our patients were younger and thinner. We focused only on the stage IA patients and provided a detailed subgroup analysis to identify the risk factors for poor outcomes in MIS and OP, though it was retrospective evidence. We were looking forward to more prospective trials targeting Asian patients to compare the outcomes of MIS and OP in EC, and further provide more accurate information for decision making for Asian women.

One of the strengths of our study is that we have used a statistical matched-paired model in both the whole population and the subgroup to minimize heterogeneity between groups, and then assess the clinical outcomes stratified by histologic type between MIS and OP in stage IA EC women. A Cochrane Database based study including a total of 4389 women in nine studies has reported no significant difference in severe postoperative morbidity, overall survival (OS) and PFS between the MIS and OP group, although MIS is linked to reduced operative morbidity and hospital stay. However, the information of histologic type is not detailed in this study [19]. Another recent retrospective study, including both low-risk and high-risk histology types, has indicated that the surgical approach does not influence the length of PFS or OS between MIS and OP after matching by homogenous groups. However, the duration of follow-up in the MIS group is almost one-year shorter than that in the OP group (50.8 ± 30.2 versus 60.6 ± 36.0 months, *p* = 0.012), which might have resulted in a bias in the amount of relapse and death [20]. In our study, although the length of follow-up is shorter in the MIS group than in the OP group, the number of the recurrence is statistically sufficient to prove that MIS is associated with a reduced PFS in stage IA low-risk EC.

Another strength of this study is that we have performed a subgroup survival analysis in stage IA low-risk histologic patients to identify the prognostic factors related to surgical approach. According to the National Comprehensive Cancer Network (NCCN) guidelines, the older age, myometrial invasion (MI) along with lymphovascular space invasion (LVSI) have been believed to deteriorate the prognosis in early-stage EC [21, 22]. One recent retrospective analysis of the U.S. National Cancer Data Base has revealed that MIS could improve OS in all elder EC women with an increased survival rate by 12% (HR = 0.86; 95%CI = 0.80–0.92; *p* < 0.001), leaving its impact on PFS unknown [23]. However, we fail to detect the superiority of MIS on PFS in stage IA elder EC patients in our study, indicating patients’ age would not influence the impact of surgical approach on PFS.

Meanwhile, our study figures out that the presence of MI would further attenuate PFS in low-risk histologic patients underwent laparoscopy. In other words, the invasion of tumor is deeper, the more pernicious it is, and the less benefit patients obtain from MIS, which to our knowledge might be attributed to both the application of uterine manipulator and the setup of intra-abdominal pressure (IAP) in MIS. The impact of the uterine manipulator on oncological prognosis remains controversial in EC [24–28]. A multi-center retrospective study consisting of 2661 women has found the manipulator would increase the recurrence and shorten the PFS in EC, regardless of it in the whole population or uterus-confined EC [25]. The manipulator is also reported to facilitate tumor cell spillage into the peritoneal cavity [27]. A recent meta-analysis also reveals a positive correlation between the use of manipulator and the malignant cytology in EC, although it indicates the manipulator would not increase the risk of recurrence and LVSI [24]. In our study, the manipulator is widely used in MIS, and our findings are consistent with above studies, showing that MIS is also associated with a higher positive peritoneal wash cytology and lower PFS in low-risk histologic patients, but not in high-risk histologic women. The limitation of our study is that the information of when the manipulator used during the surgical procedure has not been recorded, resulting in when the peritoneal wash is collected unknown.

Another issue the MIS blamed for is that the IAP, which is built commonly by CO_2_ to enlarge the operation field, would increase the metastasis of tumor, especially in the port site [29, 30]. That is the reason why gasless laparoscopic procedure, low-pressure laparoscopy, and vaginal natural orifice transvaginal endoscopic surgery (vNOTES) have been increasing adopted in the management of early-stage EC [31–34]. MIS is also thought to contribute to iatrogenic tumor spill and tumor invasion into vascular during the operation, even performed by an experienced surgeon [35, 36]. These findings are helpful to explain why the oncological outcomes in the MIS group is worse in low-risk histologic EC with MI in our study. However, the exact value of IAP is not detailed in our medical records, and further studies are needed to find out the optimum IAP in MIS. Concerning early-stage high-risk histologic tumors, in accordance with previous studies [37–39], there is no difference on survival outcomes between the MIS and OP in our study, which in turn further proves that the impact of characteristic of high-risk histology on oncological outcomes is far beyond surgical route on them.

## Conclusions

In summary, our study suggests that MIS is associated with a poorer PFS in women with stage IA low-risk histologic EC, especially those with MI. Surgical approach would not influence the oncological outcomes in women with early-stage high-risk histologic EC. Therefore, careful patient selection and surgical technique are crucial when considering MIS as a treatment option for early-stage EC patients.

### Supplementary Information

**Additional file 1:** **Table S1.** A univariate analysis for PFS in EC patients. **Table S2.** Demographics and pathology results in low-risk histologic EC stratified by myometrial invasion. **Table S3.** Demographics and pathology results in low-risk histologic EC stratified by age at diagnosis. **Table S4.** Demographics and pathology results in low-risk histologic EC stratified by lymphovascular space invasion. **Table S5.** A multivariate analysis for PFS in low-risk EC patients.

## Data Availability

The datasets used and analyzed during the current study are available from the corresponding author on reasonable request.

## Abbreviations

EC: Endometrial cancer
G3E: Grade 3 endometrioid adenocarcinoma
PS: Papillary serous carcinoma
CC: Clear cell carcinoma
CS: Carcinosarcoma
PFS: Progression free survival
MIS: Minimally invasive surgery
OP: Open surgery approach
BMI: Body mass index
FIGO: The International Federation of Gynecology and Obstetrics
DSS: Disease-specific survival
HR: Hazard ratio
CI: Confidence interval
MI: Myometrial invasion
LVSI: Lymph-vascular space invasion
IAP: Intra-abdominal pressure
