# Supplementary material for: Impact of surgical approach on progress of disease by type of histology in stage IA endometrial cancer: a matched-pair analysis
Source: BMC Surg. 2024 Jan 3;24:9. doi: 10.1186/s12893-023-02299-7 (PMC10765681; doi:10.1186/s12893-023-02299-7)
Supplement: Supplementary file 1 — Additional file 1: Table S1. A univariate analysis for PFS in EC patients. Table S2. Demographics and pathology results in low-risk histologic EC stratified by myometrial invasion. Table S3. Demographics and pathology results in low-risk histologic EC stratified by age at diagnosis. Table S4. Demographics and pathology results in low-risk histologic EC stratified by lymphovascular space invasion. Table S5. A multivariate analysis for PFS in low-risk EC patients. [file 12893_2023_2299_MOESM1_ESM.docx]

Supplementary

**Table S1**. A univariate analysis for PFS in EC patients

| **Variables** | ***P* value** | **HR (95% CI)** |
| --- | --- | --- |
| Age＜60yrs | 0.418 | 0.42(0.17-1.06) |
| BMI＞24(kg/m2) | 0.436 | 0.64(0.21-1.98) |
| Arterial hypertension | 0.310 | 1.62(0.64-4.10) |
| Diabetes mellitus | 0.334 | 0.37(0.05-2.78) |
| Menopause | 0.220 | 1.91(0.68-5.35) |
| Nulliparity | 0.495 | 21.94(0.003-1.55*10^5^) |
| MIS | 0.042 | 6.16 (1.27-29.81) |
| Lymphadenectomy | 0.077 | 0.33 (0.10-1.13) |
| Chemotherapy | ＜0.001 | 44.83 (5.96-33.99) |
| Radiotherapy | 0.086 | 2.97 (0.86-10.28) |
| MI | 0.035 | 8.77 (1.17-65.90) |
| LVSI | 0.009 | 4.44 (1.46-13.48) |
| High-risk pathology | ＜0.001 | 9.79 (3.78-25.30) |
| Positive peritoneal cytology | 0.340 | 1.44 (0.68-3.02) |

BMI, body mass index; MIS, minimally invasive surgery; MI, myometrial invasion; LVSI, lymph-vascular space invasion; HR, hazard ratio; CI, confidence interval.

**Table S2.** Demographics and pathology results in low-risk histologic EC stratified by myometrial invasion

| **Variable** | **MI (-)** | | | **MI <1/2** | | |
| --- | --- | --- | --- | --- | --- | --- |
|  | **MIS (n=151)** | **OP(n=44)** | **P value** | **MIS (n=238)** | **OP(n=101)** | **P value** |
| Age(years) | 49.52±8.70 | 54.82±8.58 | <0.001 | 53.40±8.87 | 57.04±9.40 | 0.001 |
| Arterial hypertension | 43(28.48%) | 14(31.18%) | 0.668 | 84(35.29%) | 32(31.68%) | 0.522 |
| Diabetes mellitus | 13(8.61%) | 7(15.91%) | 0.180 | 37(15.55%) | 15(14.85%) | 0.871 |
| Menopause | 48(31.79%) | 24(54.55%) | 0.006 | 142(59.66%) | 69(68.31%) | 0.133 |
| Nulliparity | 9(5.96%) | 3(6.82%) | 0.837 | 17(7.14%) | 4(3.96%) | 0.266 |
| Lymphadenectomy | 75(49.67%) | 19(43.18%) | 0.449 | 151(63.45%) | 63(62.38%) | 0.852 |
| Radiotherapy | 5(3.31%) | 1(2.27%) | >0.99 | 17(7.14%) | 4(3.96%) | 0.266 |
| Chemotherapy | 13(8.61%) | 4(9.10%) | 0.921 | 87(36.55%) | 13(12.87%) | <0.001 |
| Grade |  |  | 0.918 |  |  | 0.896 |
| G1 | 111(73.51%) | 32(27.27%) |  | 62(26.05%) | 27(26.73%) |  |
| G2 | 40(26.49%) | 12(72.73%) |  | 176(73.95%) | 74(73.27%) |  |
| Positive LVSI | 0(0.00%) | 1(2.27%) | 0.226 | 17(7.14%) | 4(3.96%) | 0.266 |
| Positive peritoneal cytology | 17(11.26%) | 4(9.09%) | 0.895 | 38(15.97%) | 5(4.95%) | 0.005 |
| Recurrence | 0(0.00%) | 0(0.00%) | -- | 7(2.94%) | 0(0.00%) | 0.108 |
| Death | 0(0.00%) | 0(0.00%) | -- | 1(0.42%) | 0(0.00%) | >0.99 |
| Length of follow-up (months) | 53.41±24.33 | 82.30±36.00 | <0.001 | 52.54±25.05 | 84.68±33.17 | <0.001 |

**Table S3.** Demographics and pathology results in low-risk histologic EC stratified by age at diagnosis

| **Variable** | **Age < 60 years old** | | | **Age ≥ 60 years old** | | |
| --- | --- | --- | --- | --- | --- | --- |
|  | **MIS (n=319)** | **OP(n=96)** | **P value** | **MIS (n=70)** | **OP(n=49)** | **P value** |
| Age(years) | 48.95±6.72 | 51.01±5.06 | 0.001 | 65.33±4.82 | 66.86±5.75 | 0.119 |
| Arterial hypertension | 93(29.15%) | 23(23.96%) | 0.320 | 34(48.57%) | 23(46.94%) | 0.861 |
| Diabetes mellitus | 32(10.03%) | 8(8.33%) | 0.621 | 18(25.71%) | 14(28.57%) | 0.729 |
| Menopause | 122(38.24%) | 45(46.88%) | 0.131 | 68(97.14%) | 48(97.96%) | ＞0.99 |
| Nulliparity | 24(7.52%) | 5(5.21%) | 0.435 | 2(2.86%) | 2(4.08%) | ＞0.99 |
| Lymphadenectomy | 189(59.25%) | 56(58.33%) | 0.873 | 37(52.86%) | 26(53.06%) | 0.982 |
| Radiotherapy | 17(5.33%) | 3(3.13%) | 0.354 | 5(7.15%) | 2(4.08%) | 0.698 |
| Chemotherapy | 85(26.65%) | 13(13.54%) | 0.008 | 15(21.43%) | 4(8.16%) | 0.052 |
| MI |  |  | 0.240 |  |  | 0.454 |
| None | 131(41.07%) | 33(34.38%) |  | 20(28.57%) | 11(22.45%) |  |
| ＜1/2 | 188(58.93%) | 63(65.63%) |  | 50(71.43%) | 38(77.55%) |  |
| Grade |  |  | 0.281 |  |  | 0.677 |
| G1 | 136(42.63%) | 35(36.46%) |  | 37(52.86%) | 24(48.98%) |  |
| G2 | 183(57.37%) | 61(63.54%) |  | 33(47.14%) | 25(51.02%) |  |
| Positive LVSI | 13(4.08%) | 5(5.21%) | 0.640 | 4(5.71%) | 0(0.00%) | 0.142 |
| Positive peritoneal cytology | 47(14.73%) | 6(6.25%) | 0.029 | 8(11.43%) | 3(6.12%) | 0.508 |
| Recurrence | 6(1.88%) | 0(0.00%) | 0.344 | 1(1.43%) | 0(0.00%) | ＞0.99 |
| Death | 1(0.31%) | 0(0.00%) | ＞0.99 | 0(0.00%) | 0(0.00%) | -- |
| Length of follow-up (months) | 52.82±24.88 | 82.29±36.13 | <0.001 | 53.16±24.30 | 87.22±29.27 | <0.001 |

**Table S4.** Demographics and pathology results in low-risk histologic EC stratified by lymphovascular space invasion

| **Variable** | **LVSI (-)** | | | **LVSI (+)** | | |
| --- | --- | --- | --- | --- | --- | --- |
|  | **MIS (n=372)** | **OP(n=140)** | **P value** | **MIS (n=17)** | **OP(n=5)** | **P value** |
| Age(years) | 51.69±8.98 | 56.49±9.29 | <0.001 | 56.47±8.22 | 52.80±5.26 | 0.361 |
| Arterial hypertension | 120(32.26%) | 43(30.71%) | 0.738 | 7(41.18%) | 3(60.00%) | 0.624 |
| Diabetes mellitus | 44(11.83%) | 21(15.00%) | 0.337 | 6(35.29%) | 1(20.00%) | ＞0.99 |
| Menopause | 177(47.58%) | 90(64.29%) | 0.001 | 13(76.47%) | 3(60.00%) | 0.585 |
| Nulliparity | 25(6.72%) | 7(5.00%) | 0.473 | 1(5.88%) | 0(0.00%) | ＞0.99 |
| Lymphadenectomy | 214(57.53%) | 79(56.43%) | 0.823 | 12(70.59%) | 3(60.00%) | ＞0.99 |
| Radiotherapy | 20(5.38%) | 4(2.86%) | 0.229 | 2(11.76%) | 1(20.00%) | ＞0.99 |
| Chemotherapy | 85(22.85%) | 13(9.29%) | 0.001 | 15(88.24%) | 4(80.00%) | ＞0.99 |
| MI |  |  | 0.040 |  |  | 0.077 |
| None | 151(40.59%) | 43(30.71%) |  | 0(0.00%) | 1(20.00%) |  |
| ＜1/2 | 221(59.41%) | 97(69.29%) |  | 17(100.00%) | 4(80.00%) |  |
| Grade |  |  | 0.303 |  |  | 0.227 |
| G1 | 173(46.51%) | 58(41.43%) |  | 0(0.00%) | 1(20.00%) |  |
| G2 | 199(53.49%) | 82(58.57%) |  | 17(100.00%) | 4(80.00%) |  |
| Positive peritoneal cytology | 52(13.98%) | 9(6.43%) | 0.019 | 3(17.65%) | 0(0.00%) | ＞0.99 |
| Recurrence | 6(1.61%) | 0(0.00%) | 0.196 | 1(5.88%) | 0(0.00%) | ＞0.99 |
| Death | 1(0.27%) | 0(0.00%) | ＞0.99 | 0(0.00%) | 0(0.00%) | -- |
| Length of follow-up (months) | 52.92±24.25 | 83.86±34.25 | <0.001 | 52.00±34.81 | 86.60±26.80 | 0.055 |

**Table S5**. A multivariate analysis for PFS in low-risk EC patients

| **Variables** | ***P* value** | **HR (95% CI)** |
| --- | --- | --- |
| MIS | 0.040 | 5.58 (1.23-25.25) |
| Chemotherapy | 0.023 | 0.08 (0.009-0.71) |
| MI | 0.076 | 0.158 (0.021-1.22) |
| LVSI | 0.174 | 0.45 (0.14-1.42) |

MIS, minimally invasive surgery; MI, myometrial invasion; LVSI, lymph-vascular space invasion; HR, hazard ratio; CI, confidence interval.
